# Role of Extracellular Heat Shock Protein 90 Alpha in the Metastasis of Oral Squamous Cell Carcinoma: A Systematic Review

**DOI:** 10.7759/cureus.38514

**Published:** 2023-05-03

**Authors:** Nishath Sayed Abdul, Najla Ahmad Alrashed, Sara Alsubaie, Hadeel Albluwi, Hessa Badr Alsaleh, Norah Alageel, Ra’ed Ghaleb Salma

**Affiliations:** 1 Department of Oral and Maxillofacial Surgery and Diagnostic Sciences, College of Dentistry, Riyadh Elm University, Riyadh, SAU; 2 Department of Dentistry, College of Dentistry, Riyadh Elm University, Riyadh, SAU; 3 Department of Oral and Maxillofacial Surgery and Diagnostic Sciences, Riyadh Elm University, Riyadh, SAU

**Keywords:** heat shock protein 90 alpha, heat shock protein, stress, metastasis, oral squamous cell carcinoma, extracellular heat shock protein 90 alpha

## Abstract

Heat shock proteins (HSPs) are expressed in a variety of cancers in human beings and are correlated with differentiation, proliferation, and metastasis. Head and neck squamous cell carcinomas, like other tumors, are exposed to environmental stress, and lack of oxygen and nutrients, and in such situations, hypoxic inducible factor (HIF) initiates the expression of genes causing angiogenesis, invasion, and metastasis. Extracellular heat shock proteins 90 alpha (eHSP90α) are overexpressed in cancers leading to tumor progression and metastasis. Hence, this review will focus on the role of eHSP90α in the metastasis of oral squamous cell carcinomas (OSCC). Different online databases were scoured for relevant articles from October 2000 to October 2022. A total of 342 articles along with duplicates were excluded. The retrieved 45 articles were studied and 39 of them were found to be not eligible as they lacked intervention and their outcome measures did not match with the present review. The final qualitative evaluation included four articles that fulfilled the eligibility criterion. A definitive expression of HSP90 was implicated, as seen in three studies, suggesting its probable role as a prognostic marker for OSCC, but no conclusive evidence was found. The present review suggests that eHSP90α plays a significant role in OSCC. Though a positive association was found between HSP90 expression and its possible correlation with metastasis, affirmative evidence can only be derived with the conduction of many more research studies and their subsequent synthesis of results.

## Introduction and background

Molecular chaperones are a family of proteins that play a crucial role in the proper folding, stability, and degradation of proteins. These proteins are also known as heat shock proteins (HSPs) because their expression is induced in response to stressors like heat shock, hypoxia, and oxidative stress [1]. There are several classes of molecular chaperones, classified by their molecular weight, structure, and function. One such class is the heat shock protein 90 (HSP90) family, which is one of the most abundant and evolutionarily conserved molecular chaperones in eukaryotes [1]. HSP90 proteins are involved in a variety of cellular processes, including protein folding and maturation, signal transduction, cell cycle control, and stress response [2]. They interact with a large number of client proteins, including transcription factors, kinases, and other molecular chaperones [2]. In addition to their role in protein folding and stability, HSP90 proteins also play a role in protein degradation by targeting misfolded or damaged proteins to the proteasome for degradation [2].

Given their important role in protein folding and stability, it is not surprising that HSP90 proteins have been implicated in a variety of diseases, including cancer, neurodegenerative disorders, and cardiovascular disease [3]. In cancer, HSP90 has been identified as a potential therapeutic target because it is overexpressed in many tumor types and is essential for the stability and function of many oncogenic proteins [3]. Inhibition of HSP90 has been shown to induce degradation of these client proteins and lead to tumor cell death [4]. The HSP90 family of molecular chaperones is an important player in maintaining proper protein folding, stability, and degradation in cells. Their roles in cellular processes and disease make them an attractive target for therapeutic intervention [5]. These proteins were first discovered by Ferruccio Ritossa, an Italian scientist, in 1962, who supports the hypothesis that when a cell is subjected to environmental stress, it undergoes a response in the form of HSPs [6]. The consequent activation of corresponding genes to heat was linked to the elevation of a particular protein being expressed [7]. HSPs have become a subject of interest in several studies due to their universal presence in all living things and their role as chaperones in the maintenance and proper folding of client proteins, since their identification [8-11].

HSPs are categorized into five main groups based on their molecular weight, shape, and function: HSP 100, 90, 70, 60, and the small HSP (sHSP)/crystallins [9]. The overexpression of HSP90 has been linked to disorders such as cancers, viral infections, inflammation, and neurological diseases, indicating that HSP90 may aid in the development of oral cancer [10]. HSP90 proteins exist in the cytoplasm and are involved in regulating the folding, stabilization, and activation of a variety of signaling molecules and oncogenic client proteins [11]. HSP 90 alpha (HSP90α) is a subtype of HSP90 that is frequently overexpressed in cancer cells and is associated with a more aggressive phenotype [12]. The overexpression of HSP90α has been observed in several types of cancer, including oral squamous cell carcinoma (OSCC) [8]. The expression of HSP90α in OSCC is often associated with poor prognosis and is considered a potential therapeutic target [11]. The expression pattern of HSP90α proteins has been extensively studied in OSCC, and several studies have demonstrated that HSP90α is overexpressed in OSCC tissues compared to normal tissues [13]. Additionally, the overexpression of HSP90α has been shown to be associated with lymph node metastasis, advanced tumor stage, and poor survival in OSCC patients [14]. These findings suggest that HSP90α may play a critical role in the progression and metastasis of OSCC and may serve as a potential diagnostic and therapeutic target in this disease.

Very limited studies were done previously to rule out the possible role of extracellular HSP90α (eHSP90α) in the metastasis of oral cancer [11-14]. Therefore, the present review aimed to analyze the role of HSP90α in the metastasis of OSCC, which will help to determine their role as prognostic markers of cancer metastasis and thereby, can target HSP90, when considering its therapeutic use in OSCC.

## Review

Research question

Does extracellular heat shock protein 90 alpha (eHSP90α) play any significant role in the metastasis of oral squamous cell carcinoma?

The research question was framed following the PICO (population (P), intervention (I), comparison (C), and outcome (O)) statement as follows: the patients diagnosed and confirmed with OSCC (P), presence of HSP90α in OSCC cases (I), compared with healthy controls/normal tissue (C), and outcome measures (secondary) include the prognostic factors, survival rate, and psychological status of patients with metastatic OSCC.

The study was in accordance with the Preferred Reporting Items for Systematic Reviews and Meta-Analyses (PRISMA) protocol for guidelines pertaining to the conduction of systematic reviews like these [15].

Literature search

For this systematic review, the reviewers formulated a comprehensive search strategy to identify relevant studies across six major databases, namely, MEDLINE (PubMed), the Cochrane Library, EBSCOhost (Dentistry & Oral Sciences Source), Embase, ScienceDirect, and Google Scholar. The search was conducted from October 2000 to October 2022 to capture studies published in the last 22 years. The search strategy was developed using a combination of Medical Subject Heading (MeSH) terms and Boolean operators.

In MEDLINE (PubMed), the reviewers used a combination of keywords, including "oral squamous cell carcinoma," "heat shock proteins," and "metastasis." In addition, they used MeSH terms such as "HSP90 heat-shock proteins" and "neoplasm metastasis." The search strategy also included Boolean operators such as "AND," "OR," and "NOT" to narrow down the search results.

Similarly, for the Cochrane Library, the reviewers used a combination of keywords, including "oral cancer," "HSP90 alpha," and "prognosis." The search strategy was formulated using the PICO strategy to identify relevant studies. The search was limited to randomized controlled trials and systematic reviews. For EBSCOhost (Dentistry & Oral Sciences Source), the search strategy was developed using a combination of keywords and MeSH terms, including "oral squamous cell carcinoma," "HSP90 heat-shock proteins," and "prognosis." In Embase, the search strategy included a combination of keywords such as "oral cancer," "heat shock proteins," and "lymph node metastasis." MeSH terms such as "HSP90 heat-shock proteins" and "neoplasm metastasis" were also used. The search was limited to studies published in the last 22 years. For ScienceDirect, the reviewers used a combination of keywords including "oral squamous cell carcinoma," "HSP90 alpha," and "prognosis." MeSH terms such as "HSP90 heat-shock proteins" and "neoplasm metastasis" were also included.

Finally, for Google Scholar, the reviewers used a combination of keywords including "oral cancer," "HSP90 alpha," and "metastasis." The search strategy also included the use of Boolean operators such as "AND," "OR," and "NOT" to narrow down the search results. However, due to the vast number of search results, the reviewers only included studies that were available in full text and published in the English language.

Eligibility criteria for the studies

Specific criteria for study selection were utilized in this systematic review. The inclusion criteria consisted of studies that reported the expression of HSP90α in OSCC or normal oral mucosa, studies that evaluated the association between HSP90α expression and OSCC clinicopathological features, studies that investigated the role of HSP90α in OSCC metastasis, studies that reported the prognostic value of HSP90α expression in OSCC, and studies that investigated the biological function and mechanism of HSP90α in OSCC. In contrast, the exclusion criteria consisted of studies that were not published in English, studies that were not conducted on human samples, reviews, letters, conference abstracts, case reports, and studies with insufficient data to extract or calculate hazard ratios, odds ratios, or mean values. The reviewers performed a comprehensive literature search using relevant keywords and MeSH terms to identify potentially relevant studies. Then, they screened the titles and abstracts of the retrieved studies and assessed their eligibility based on the inclusion and exclusion criteria. Finally, they assessed the full-text articles of the selected studies and extracted relevant data. The strict study selection criteria employed by the reviewers ensured the inclusion of high-quality studies with a low risk of bias, which enhanced the validity and reliability of the findings of this systematic review.

Study selection

The reviewers formulated a search strategy for this review by combining relevant keywords and MeSH terms. The search was conducted in electronic databases, including PubMed, Embase, and Cochrane Library. The search was limited to articles published in the English language between 2000 and 2021. The search strategy aimed to identify all relevant articles on HSP90α expression in OSCC and its association with prognosis and lymph node metastasis.

After the initial search, duplicates were removed, and titles and abstracts were screened for eligibility. The PICO strategy was employed to formulate the research question and guide the study selection process. Full-text articles were then reviewed for eligibility based on predetermined inclusion and exclusion criteria. The reviewers faced challenges in identifying relevant articles due to the heterogeneity of study designs, patient populations, and outcomes. To address discrepancies in study selection, the reviewers discussed and resolved any disagreements through consensus. Additionally, reference lists of included articles were manually searched to identify any relevant studies missed in the initial search. The final selection of studies included in this systematic review was based on the study selection criteria and search strategy outlined above. Overall, the search strategy and PICO strategy were effective in guiding the study selection process and identifying relevant articles for this review.

This systematic review could not be converted into a meta-analysis due to significant heterogeneity in the included studies regarding the methods and results. The studies varied in terms of patient demographics, sample size, HSP90α detection methods, and outcome measures. Some studies measured the expression of HSP90α in tumor tissues, while others measured it in serum or saliva. Additionally, the cut-off values for HSP90α expression varied among the studies. These factors made it challenging to pool the data from the included studies for meta-analysis, and therefore, the reviewers opted for a narrative synthesis of the findings.

Ethical clearance

Ethical clearance for the study was obtained from the Review Board of Riyadh Elm University (IRB number: FUGRP/2022/297/872/816).

Risk of bias evaluation

The reviewers screened the titles and abstracts based on eligibility criteria. Any conflict arising between the two was resolved by a third reviewer. Full-text articles of the selected studies were then obtained and evaluated. Each article was looked for characteristics of the study, location of the study, examination site, technique, evaluator, and findings. Methodological quality evaluation of included studies was done. The Newcastle-Ottawa Scale (NOS) was employed to find the quality of articles. The studies scored above 6, thus ensuring a lower risk of bias.

**Figure 1:**
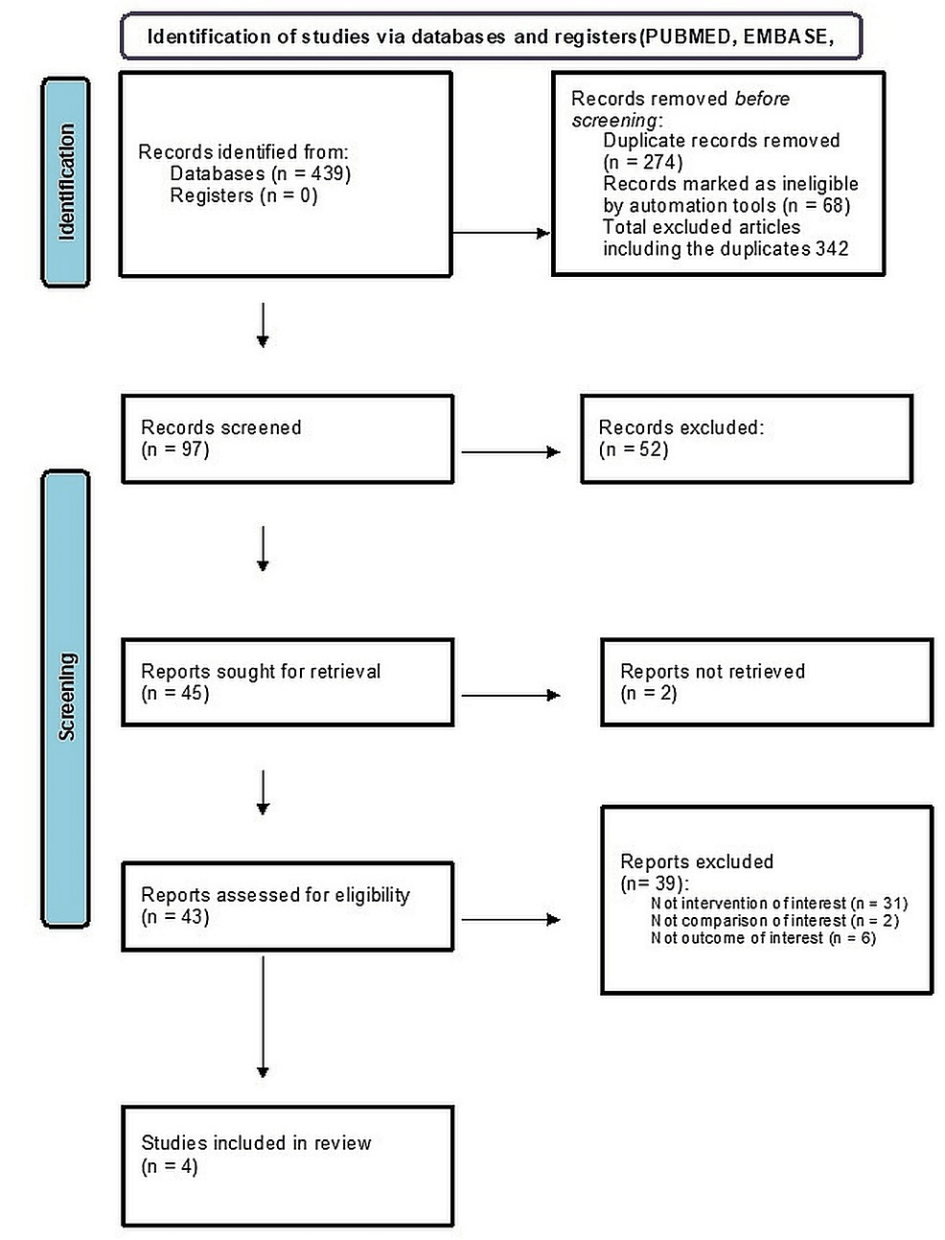
PRISMA flow chart for the review PRISMA: Preferred Reporting Items for Systematic Reviews and Meta-Analyses.

A thorough literature search identified a total of 439 manuscripts. Out of these, 274 were duplicate records and 68 were not following the eligibility criteria that were identified through Endnote X8 software (Clarivate, London, UK) and, therefore, were excluded. A total of 342 articles along with duplicates were excluded. Around 92 manuscripts were screened, of which 52 were excluded as they were available as abstracts only. The retrieved 45 articles were studied and 39 of them were found to be not eligible as they lacked intervention and their outcome measures do not match with the present review. The final qualitative evaluation included four articles that fulfilled the eligibility criterion. Manual searches did not yield any articles. Figure 1 presents the literature search process as per PRISMA guidelines.

The present review could not be converted to a meta-analysis as the included articles did not provide the required quantitative data for analysis, and due to heterogeneity of the available data. Therefore, this study is only a systematic review. Out of four articles that are included in the present review, three studies were done on tissues and one on cell lines. Two studies were from Taiwan, while two others were from Japan. The results of data extraction are presented in Table 1.

**Table 1:** Characteristics of studies included in the review OSCC: oral squamous cell carcinoma; HSP: heat shock protein; HSP90: heat shock protein 90.

Author	Location	Examination site	Technique used	Evaluator	Result/findings
Chang et al. (2017) [16]	Taiwan	Tissues from 36 mongoloid patients newly diagnosed with OSCC between 20 and 65 years	Avidin-biotin technique of immunohistochemical staining	Single pathologist	HSP90 expression was significantly elevated in the tumor samples in comparison to normal sites at p < 0.001. Lymph node metastasis was found in 16 of the 36 dissected cases
Shiraishi et al. (2021) [17]	Japan	OSCC cells were obtained from tongue tissues in patients aged 30-86 years between January 2009 and March 2014. 10 normal mucosal tissues were also examined from healthy sites of the same patients	Proteomic profiling	Two specialists independently scored cases and were blinded to the clinical grading of the lesion	Molecular profiling was extracted with the aid of KeyMolnet software, which demonstrated HSP90 pathways to have the highest score (47.28) of the five pathways detected. HSP90 was hypothesized as a "target protein" playing a significant role in OSCC cell multiplication and survival HSP90 expression was significantly associated with cervical lymph node metastasis at p = 0.015
Huang et al. (2010) [18]	Taiwan	2 cell lines (OC2 - low malignancy and OCSL - high-grade malignancy) were obtained from 2 male patients affected with OSCC. The comparison was with global proteomic analysis	Immunohistochemical analysis and mass spectrometry peptide sequencing using an imaging densitometer	Single pathologist	HSP90 was significantly expressed in high-grade OSCC cases. Differential expression of proteins was noted based on the severity of the lesion
Ito et al. (1998) [19]	Yokohama	24 tissues of invasive oral squamous cell carcinoma of the tongue region	HSP immunostaining	Two pathologists	Staining of the cytoplasm of suprabasal tumor cells was noted in 17/24 samples and was positive for all dysplastic lesions. Lymph node metastasis was not correlated to HSP, with 11 tissues demonstrating metastasis in 15 negatively stained for P53 immunostaining and 6 in positively stained specimens. HSP immunostaining with lymph node metastasis showed no statistical significance

HSP90 expression in oral cancer

As seen from the above articles, a definitive expression of HSP90 was implicated as seen in three studies [16-18], suggesting their probable role as a prognostic factor with OSSC, but no conclusive evidence was found. But this result has to be exercised with caution as the co-expression of other HSPs masks the specificity of the diagnostic role in OSCC. Carcinoma of the tongue and oral mucosa were two carcinomas where HSP90 was overexpressed than OSCC. Also, their expression in a variety of malignancies further questions the diagnostic accuracy. Patients who were selected under the respective studies varied in their surgical assessment from a period of five years [17] to seven years [19], with the remaining two studies not delving into this information [16,18].

Lymph node metastasis

It can be regarded as a prognostic factor for OSCC. Two studies [16-17] reported a correlation of OSCC metastasis with HSP90 expression. Whereas, the study by Ito et al. (1998) [19] did not show any correlation of lymph node metastasis to HSP90α expression.

Heterogeneity

The studies mentioned in the table exhibit heterogeneity in several aspects. First, the location of the studies varies, with three studies conducted in Taiwan and one in Yokohama, Japan. The examination site also differs across the studies, with one study using tissues from patients diagnosed with OSCC, two studies using OSCC cells obtained from tongue tissue, and one study using two cell lines obtained from two male patients affected with OSCC. The techniques used to evaluate the expression of HSP90 also differ, with one study using the avidin-biotin technique of immunohistochemical staining, one using proteomic profiling, and two using HSP immunostaining. Additionally, the number of evaluators and their expertise in the field of study vary across the studies. Lastly, the results and findings of the studies also differ, with some studies finding a significant association between HSP90 expression and lymph node metastasis, while others find no correlation.

Discussion

This systematic review is significant in shedding light on the potential role of HSP90α as a prognostic marker for OSCC. By synthesizing the findings of several studies, the review indicates a strong correlation between HSP90α expression and the risk of lymph node metastasis and poor prognosis in OSCC patients. The review also highlights the need for caution in interpreting the diagnostic role of HSP90α in OSCC due to its co-expression with other HSPs and expression in other malignancies. One potential gap in the literature that the review addresses is the inconsistency in the findings of previous studies on the role of HSP90α in OSCC. By providing a comprehensive and critical analysis of the available literature, the review helps to clarify the existing evidence on this topic and identifies areas for future research. The findings of the review are significant in providing a better understanding of the molecular mechanisms underlying the metastasis of OSCC and the potential role of HSP90α as a prognostic marker. The review also has important implications for clinical practice, as it suggests that targeting HSP90α could be a potential strategy for preventing or treating OSCC metastasis. Overall, the systematic review contributes to the existing literature on the role of HSP90α in OSCC and provides important insights into its diagnostic and prognostic value. The findings of the review have the potential to impact future research and clinical practice in this field.

Recent literature studies [20-22] suggest that HSP90 is one of the vastly researched and abundantly found proteins of eukaryotic cells making up to 1-2% of total proteins under non-stress situations. Despite being a common housekeeping protein that is widely distributed in healthy cells, these variations make HSP90 a target protein in cancer therapy. HSP90 plays a vital role in the folding of at least 200 distinct proteins of various signaling pathways, and in the refolding of denatured proteins after stress [23,24]. HSP90 interacts with and stabilizes a growing number of different kinases, including numerous critical members of malignant transformation, such as the ErbB2, Src, Abl, or Met tyrosine kinases, or the Raf, Akt, and cyclin-dependent serine kinases. HSP90 client proteins have been demonstrated to be critical for the development, proliferation, and survival of numerous forms of cancer. Hence decreasing the activities of HSP90 has anti-cancer potential. The N-terminal domain contains a relatively unique adenosine triphosphate-binding site, and the Bergerat fold and more recently identified C-terminal domain opened new possibilities for the suppression of this chaperone to target the tumor [25,26]. Additionally, it has been demonstrated that the tumor-suppressor transcription factor p53 accumulates as a result of HSP90 binding [27,28].

A malignant behavior in cancer is characterized by the spread of tumor cells. HSP90 production contributes to the tumor cells' increased invasiveness, which is a crucial precursor to metastasis. Matrix metalloproteinase-2 (MMP-2) is activated when HSP90, which is produced by invasive cancer cells via exosomes, is present. Regarding HSP90's role in cancer malignancy, current studies contend that the release of HSP90-containing exosomes by invasive cancer cells may improve the motility of tumor cells [29].

HSPs have been shown to have prognostic and predictive value in oral cancer. HSPs are molecular chaperones that are involved in protein folding, degradation, and transport and are expressed in response to cellular stressors such as heat, hypoxia, and inflammation [27]. Several studies have investigated the expression of HSPs in oral cancer and their association with patient outcomes [16-19,28]. One of the most studied HSPs in oral cancer is HSP90α. High expression of HSP90α has been associated with poor prognosis in patients with OSCC. The expression pattern of HSP90α proteins has been extensively studied in OSCC, and several studies have demonstrated that HSP90α is overexpressed in OSCC tissues compared to normal tissues. Additionally, the overexpression of HSP90α has been shown to be associated with lymph node metastasis, advanced tumor stage, and poor survival in OSCC patients. These findings suggest that HSP90α may play a critical role in the progression and metastasis of OSCC and may serve as a potential diagnostic and therapeutic target in this disease.

In addition, HSP90α has been found to be involved in the development of drug resistance in OSCC, which is a major challenge in cancer treatment, with trastuzumab being one of the drugs whose action is diminished by HSP90 [29]. HSP90α has also been suggested to be involved in the pathogenesis of oral lichen planus (OLP), a chronic inflammatory disorder of the oral mucosa [30]. Its overexpression has been found in OLP lesions and may contribute to the inflammatory response and tissue damage [27]. Furthermore, HSP90α has been shown to play a role in the immune response in periodontal disease, a chronic inflammatory condition of the periodontium [8]. Its expression has been found to be upregulated in periodontal tissues and may contribute to the inflammatory response and tissue destruction [6]. Thus, HSP90α may have significant implications for the diagnosis, prognosis, and treatment of various oral diseases and conditions.

Since elevated levels of HSF1 are linked to oncogenesis, it is critical to find ways to use HSP90 inhibitors alongside strategies to lessen HSF1's adverse consequences. The inducible version of heat shock protein 70 (HSP70), which can promote oncogenesis in tumors, is upregulated by elevated HSF1 as well. Since cancers significantly overexpress the inducible version of HSP70 rather than the constitutive form, targeting HSP70 has been found to be beneficial [31].

The staging of oral cancer is based on the TNM (tumor, node, and metastasis) system, which considers the size and extent of the tumor (T), whether it has spread to nearby lymph nodes (N), and whether it has metastasized (M) to other parts of the body [8]. At stage 0, abnormal cells are found on the surface layer of the oral tissue, without invasion into deeper tissues. This stage is also referred to as carcinoma in situ, and it is considered a pre-cancerous stage that may progress to invasive cancer if left untreated. At stage I, the tumor is less than or equal to 2 cm in size, and it has not spread to nearby lymph nodes or distant sites. At stage II, the tumor is between 2 and 4 cm in size and has not spread to nearby lymph nodes or distant sites. At stage III, the tumor is larger than 4 cm in size or has spread to a single lymph node on the same side of the neck as the primary tumor that is less than 3 cm in size. At stage IV, the tumor is further divided into two sub-stages: at stage IVA, the tumor has grown into nearby structures such as bone, muscle, or skin, or it has spread to a single lymph node on the same side of the neck as the primary tumor that is between 3 and 6 cm in size. At stage IVB, the tumor is spread to multiple lymph nodes on the same side of the neck, lymph nodes on both sides of the neck, or distant sites. This stage is considered advanced, and treatment options may include surgery, radiation therapy, chemotherapy, or a combination of these, depending on the patient's overall health and other factors. The stage of oral cancer is an important factor in determining the appropriate treatment plan and prognosis for patients [8]. In early-stage oral cancer, HSP expression can help identify patients at high risk of recurrence or metastasis. In advanced-stage oral cancer, HSP expression can be used as a target for therapy, as inhibition of HSP expression can enhance the effectiveness of chemotherapy or radiotherapy [31]. HSP has also been implicated in other types of cancers. One study investigated the effects of overexpression of HSP70 on colon cancer and melanoma in rodent models. The study highlighted the potential interest of targeting the HSP70 interaction with other cells for cancer therapy [31]. Another study demonstrated the application of an HSP90 inhibitor in preclinical models of thyroid cancer. The results showed that HSP90 inhibition significantly reduced cell viability and induced apoptosis in thyroid cancer cell lines [22].

Our review results are compliant with a meta-analysis conducted in a study [32] wherein no significant risk was demonstrated for HSP90 with OSCC risk (0.94 (0.04 - 24.57) at p > 0.05). There is a paucity of literature regarding this context, which limits our review to only four studies, all conducted on Asian ethnicity. But the merits of this review overpower limitations in terms of good quality methodological study design and literature search not confining to any particular location or duration. To the best of our knowledge, this is the first review to synthesize the effect of HSP90 on OSCC risk and metastasis.

Although this systematic review provides valuable insights into the role of eHSP90α in the metastasis of OSCC, it is not without limitations. One potential limitation is the inclusion of only English language articles, which may have excluded relevant studies published in other languages. Additionally, the inclusion criteria only considered studies that reported on the association between eHSP90α and OSCC metastasis, which may have excluded studies that reported on other aspects of HSP90α in OSCC. Another limitation is the heterogeneity in the methods used in the included studies, including variations in the types of samples analyzed, the methods of HSP90α detection, and the definitions of metastasis. These variations could have affected the consistency and comparability of the results across the studies. Furthermore, the quality of the included studies varied, with some studies having small sample sizes, inadequate control groups, or incomplete information on patient characteristics. These limitations could have affected the strength and reliability of the evidence presented in this review. So, while this systematic review provides important insights into the role of eHSP90α in OSCC metastasis, the limitations of the included studies should be considered when interpreting the findings. Further studies with standardized methods and larger sample sizes are needed to confirm and expand upon these results.

## Conclusions

Our review suggested that HSPs indicate a significant role in OSCC in terms of cancer progression and metastatic indicators. The differentiation of HSP to oral carcinogenesis makes it a potential biomarker for its identification and aggressiveness. Though a positive association was found between HSP90 expression and its possible correlation with metastasis (mostly confined to stage IV), affirmative evidence can only be derived with the conduction of many more types of research and their subsequent synthesis of results. We further recommend global molecular studies with a larger sample to provide a definitive conclusion.
